# Facile sulfation of cellulose *via* recyclable ternary deep eutectic solvents for low-cost cellulose nanofibril preparation†

**DOI:** 10.1039/d2na00769j

**Published:** 2022-11-21

**Authors:** Guangrui Ma, Zhiguo Zhang, Jiachuan Chen, Guihua Yang, Ming He

**Affiliations:** a State Key Laboratory of Biobased Material and Green Papermaking, Qilu University of Technology (Shandong Academy of Sciences) Jinan Shangdong Province 250353 People's Republic of China heming8916@qlu.edu.cn

## Abstract

Here we present a new method to treat cellulose with a sulfamic acid–urea–choline chloride (ternary deep eutectic solvent) system, which can realize both swelling and sulfation of cellulose. This can greatly reduce the energy consumption in the process of cellulose nanoization, and use it to successfully prepare food packaging films for eliminating odors. We hope that due its simplicity and resource-efficiency, this method will have a widespread influence on currently used (nano) cellulose modification protocols.

## Abstract 2

DESs can swell and sulfate cellulose, greatly reduce the production energy consumption of CNF, and successfully used CNF in food packaging film.

## Graphical abstract

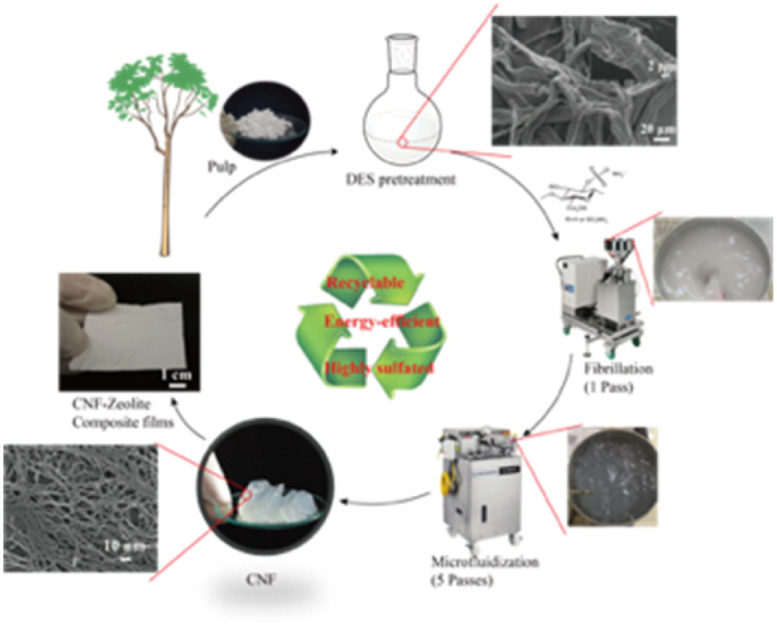

In the field of sustainable materials and chemistry, the production and separation of nano-cellulose fibers from natural cellulose fibers have gained significant scientific and industrial interest in recent years.^1^ One type of nano-cellulose fibers is cellulose nanofibrils (CNFs), which when compared with their micro-sized counterparts, exhibit improved performance, including significantly higher stiffness and strength and enlarged surface area. The unique intrinsic properties of nanocellulose can lead to the production of new materials,^2^ such as flexible and lightweight green electronic products and even recyclable solar cells.^3,4^

Cellulose is widely available as the structural material of plants, where it already exists as nanosized fibers. However, these nanocellulose fibers (elemental fibrils) held larger fiber bundles together, which are combined by strong hydrogen bonds and weak van der Waals forces. Strong mechanical force can break the natural and stubborn fiber structure and release CNF.^5^ Unfortunately, overcoming the forces that resist fibril disassembly inevitably means high input of mechanical energy and thus high energy demand. As a way out, several chemical methods have been introduced.^6^ Generating strong surface charge on natural fibers is the most effective method to produce high-quality CNFs.^7^ Chemical modifications such as (2,2,6,6-tetramethylpiperidin-1-yl)oxyl (TEMPO) mediated oxidation,^8^ carboxymethylation,^9^ and periodate oxidation followed by further derivatization^10,11^ have been utilized to produce high-quality CNFs. Although these chemical modifications allow CNF to be produced with minimal mechanical force, they usually use dangerous halogenated chemicals and seriously damage cellulose fibers (*i.e.*, cause a decrease in the polymerization degree [DP] and a loss in yield).^12^ Enzymatic treatments are considered as a sustainable method to produce CNF. Enzymes cause mild hydrolysis of cellulose (*i.e.*, reduce the degree of polymerization of cellulose), which in turn allows the release of CNF while reducing energy consumption.^13^ At the same time, enzymes have long pretreatment time, high price and cannot be recycled.

More recently, non-modifying methods (no or only minimal decrease in DP or alteration of the cellulose structure) based on deep eutectic solvents (DESs) have been used for the sustainable production of CNFs.^14,15^ In addition to the use of DESs as non-modifying pretreatment media, they can be harnessed as solvents for chemical derivatization^16^ that can even be recycled^12^ in CNF production. It was shown that the nonchemical modification of cellulose fibers with DESs is assumed to cause fiber swelling.^17^ DES pretreatment composed of choline chloride and urea is one of the most representative DESs,^15^ which facilitates fibrillation into CNF. However, a choline chloride urea system will give off an unpleasant (non-modifying pretreatment) odor during pretreatment. In terms of energy consumption, compared with modification treatment, DES treatment may require higher energy consumption. This is because the interaction between fibers is reduced by hydroxyl modification.^5^

Here we would like to communicate a one-step method, where a sulfamic acid–urea–choline chloride system was used to pretreat raw fiber materials, which not only realized the swelling of fibers, but also sulfated the fiber. The approach was straightforward, and the solvent could be recycled. It should be noted that the DES could be made into a clear and transparent liquid at a lower temperature, and the temperature of the sulfate modified cellulose is only 100 °C compared with 150 °C adopted by Sirviö *et al.*^18^ We further analysed the effect of the modification on the physical properties of cellulose, as well as the energy consumption in the process of nanocrystallization, by comparing the original fiber samples and samples pretreated by DESs (pulp samples were named 1-3-1-0.5 h, 1-3-1-1 h and 1-3-1-2 h according to the pretreatment time). Finally, we studied the recovery rate of DES. Then, its application in deodorization was studied, and we found that zeolite–cellulose composite nanofibril films are suitable for capturing highly volatile sulfur containing compounds emanating *e.g.* from the foul-smelling durian fruit.

Fig. 1 shows the preparation process of sulfated CNFs: aminosulfonic acid, urea and choline chloride were mixed in a molar ratio of 1 : 3 : 1, and then stirred and heated in an oil bath at 60 °C with a magnetic stirrer. After obtaining a transparent liquid (about two hours), cellulose (the mass ratio of cellulose to the reaction system is 4% w/w) was added to DES, and then, the temperature of the system was increased to 100 °C (the heating rate is about 5 °C min^−1^) under magnetic stirring, and the pretreatment time was 30, 60, and 120 minutes, respectively. The reaction mixture was taken out of the oil bath, cooled for 5 min, excess water was added, the reaction was terminated, and it was filtered and washed with water until the filtrate was neutral. The washed fibers were collected and stored at 4 °C until the next use. The yield after slurry treatment is 80–99%, and the loss in yield was mainly caused by sample treatment (such as a small amount of fiber left on the filter paper).

**Fig. 1:**
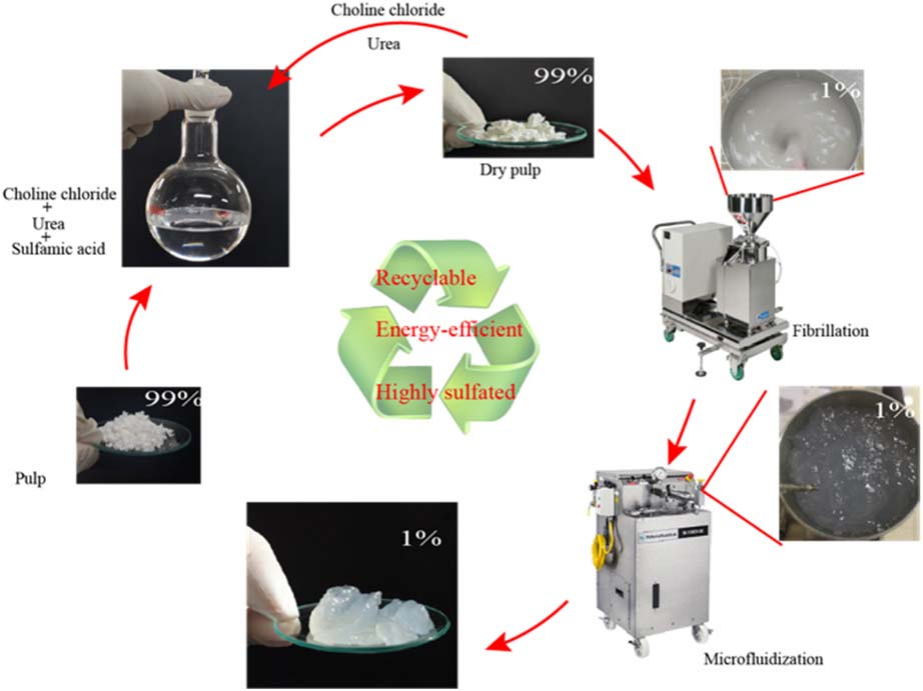
Preparation process of sulfated CNFs.

The quality of the fiber was directly analyzed from undried samples to prevent the fiber from shrinking. The raw pulp is soaked in deionized water for the same time, and the results are shown in Tables 1 and S1.† The fiber width, length (Lc(*w*)) and curl were obtained as average values of over 5000 individual qualified fibers captured by using a fiber image analyzer.^12^ It was found from Tables 1 and S1† that the width of fibers pretreated by DESs increases from 13.71 μm to 14.04 μm, and the curl decreases from 34.59% to 6.12%. In DES pretreated samples, with the extension of treatment time, the fiber width increases, the entanglement degree decreases, and the fiber length decreases, and the DP of fiber decreased gradually with the prolongation of pretreatment time. This may be mainly because the interaction between fibers based on hydrogen bonds is weakened by DES pretreatment.^19,20^

**Table 1:** Physical properties of fibers

	Fiber length/mm	Fiber width/μm	Curl/%	DP
Pulp	0.76	13.71	34.59	1211
1-3-1-2 h	0.52	14.04	6.12	707

Fig. 2 shows scanning electron microscope images of fiber samples. As shown in Fig. 2, it could be seen from Fig. 2a(i) that the fiber surface of the original pulp was relatively smooth, and there were no raised small fibers. After DES pretreatment, some fine fibers appeared on the fiber surface, as shown in Fig. 2b(i). Comparing Fig. S1a(i) and b(i)† and 2b(i), it could be found that the longer the pretreatment time of DES slurry, the rougher the fiber surface was.

**Fig. 2:**
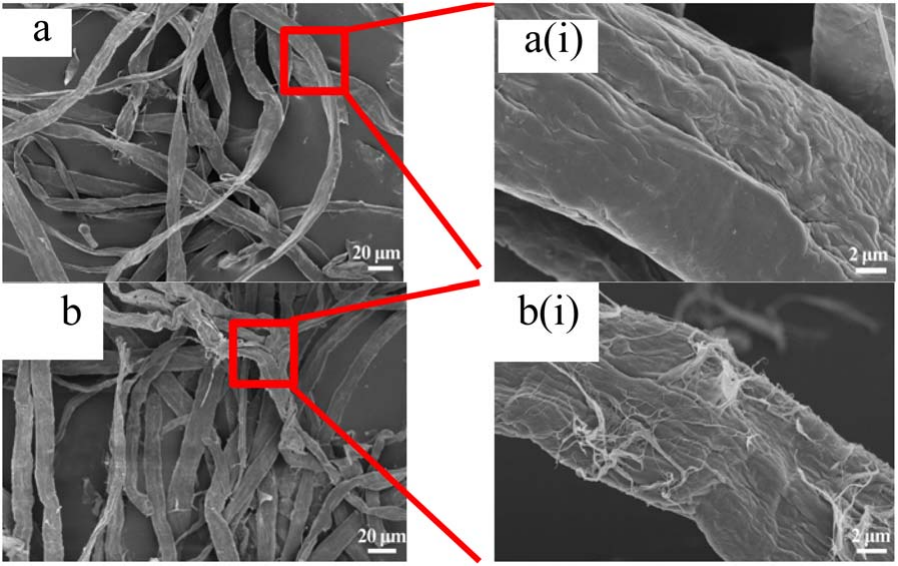
Scanning electron micrograph of fiber. (a) Pulp and (b) 1-3-1-2 h.

Chemical modification of cellulose could be observed in the infrared spectrum in Fig. 3. Asymmetric S=O and symmetric C–O–S vibrations of sulfate groups appear at around 1256 cm^−1^ and 815 cm^−1^,^21,22^ respectively. This indicates that sulfonic acid groups were grafted onto cellulose. Due to the existence of sulfate, a weak peak of NH_4_^+^ was observed at 1510 cm^−1^.^23^ In addition, the stretching vibration of C=O can be observed at 1720 cm^−1^, which may be caused by the reaction between urea and cellulose to form carbamate.^24,25^

**Fig. 3:**
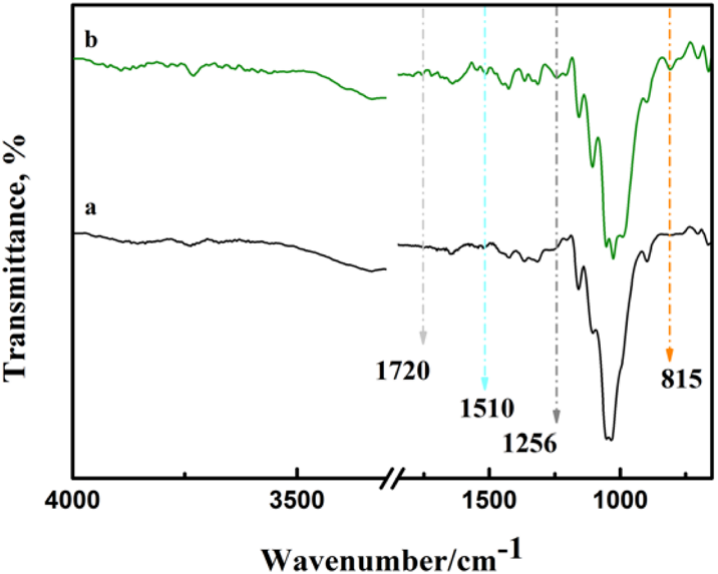
Infrared spectra of samples. (a) Pulp and (b) 1-3-1-2 h.

CNF with a sulfonate group was previously produced by periodate oxidation and then adding bisulfite. By periodate oxidation and bisulfite addition in turn, the sulfonate group content reaches 0.18–0.5 mmol g^−1^.^11,26^ Recently, sulfoethylation has been used to produce CNF with a charge density of 0.67 mmol g^−1^.^27^ Compared with previous results in the literature, the charge density of sulfated samples produced here were 0.25–0.80 mmol g^−1^ (Table S2†), which is a potential raw material for CNF with high charge density.

Fig. 4 and S2† show an atomic force microscope diagram of CNF samples prepared from pulp fibers. It could be found from Fig. 4a that there were some incompletely fibrillated fiber bundles in CNF samples, and the diameter of these fiber bundles was about 50 nm, and the diameter of CNFs was mostly between 30 and 45 nm while the fibrillation effect of DES samples shown in Fig. 4b (S2a and b†) was better, and the diameter of CNFs was mostly between 10 and 25 nm (25–40 nm, 15–35 nm).

**Fig. 4:**
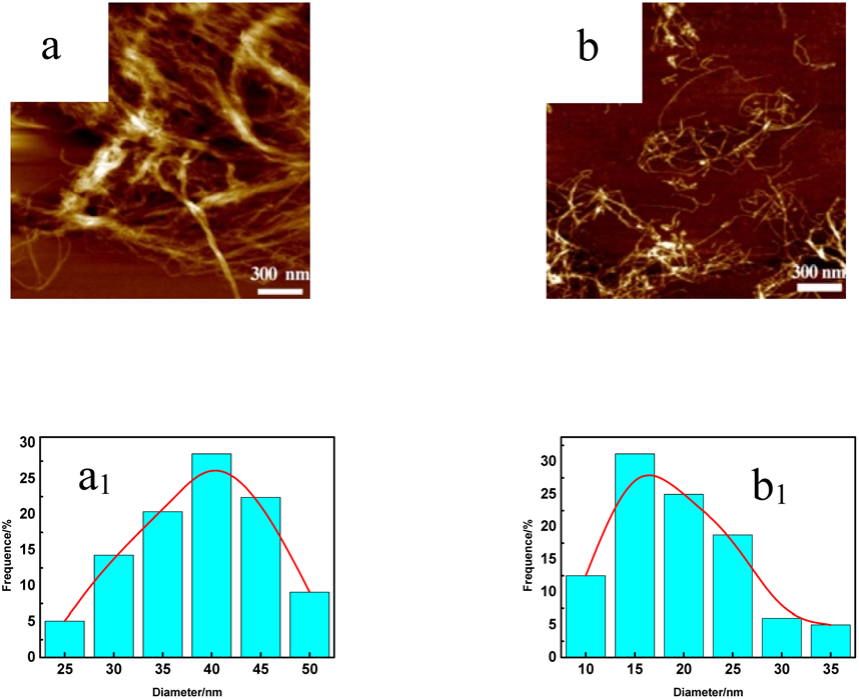
Atomic force microscopy of CNF samples. (a) CNF and (b) 1-3-1-2 h.

Fig. 5 and S3† show thermal stability spectra of the CNF samples. As shown in Fig. 5, between 35 °C and 150 °C, due to the evaporation of water in the sample, the mass of the experimental sample decreases. According to the TG curve and DTG curve shown in Fig. 5, the initial degradation temperature of CNF was 272 °C, and the temperature on reaching the maximum thermal degradation rate was 342 °C. According to the spectrograms in Fig. 5 and S3,† the initial degradation temperature of CNF prepared after DES pretreatment was 215–230 °C, and the maximum thermal degradation rate was 232–245 °C. After the end of sample pyrolysis, the change of mass fraction tends to be constant and does not change with the increase of temperature, that was, the remaining part was coke residue. The mass percentages of coke residues in CNF, 1-3-1-0.5 h, 1-3-1-1 h and 1-3-1-2 h are 16.1%, 23.6%, 25.5% and 25.51%, respectively. According to the thermal stability analysis, the longer the pretreatment time of DES, the worse the thermal stability of the prepared samples.

**Fig. 5:**
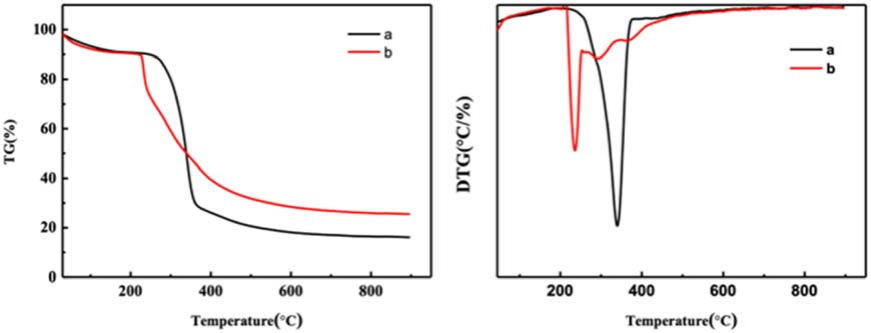
Thermal stability spectrogram of CNF samples. (a) CNF and (b) 1-3-1-2 h.

The destabilization kinetics of CNF was analyzed by TurbiSoft LAB. Due to inevitable sedimentation of nanocellulose in water, the Turbiscan Stability Index (TSI) of the water suspension of CNFs increases with time, which indicates that the dispersed phase settles faster.^28–30^Fig. 6 and S4† show the backscatter spectrum of CNF samples. From Fig. 6a, it could be found that the transmission variation of the transmission (ΔBS) value was less than 0, which indicates that CNF samples prepared from slurry without DES pretreatment have a serious agglomeration phenomenon. Compared with Fig. 6b, it could be found that the ΔBS value of the sample prepared after DES pretreatment fluctuates only a little, which indicates that the sample has good stability. It should be noted that after DES pretreatment, the prepared CNF was nano-sized, that is, it was translucent and colloidal, and some bubbles will inevitably appear in the sample preparation process, which will affect the measurement. Through the backscattering spectrogram, we can only guide the agglomeration of CNF in samples, but we cannot compare the stability of samples concretely. Therefore, the TSI of the sample was measured, and the stability of the sample can be compared by comparing the TSI value of the sample.

**Fig. 6:**
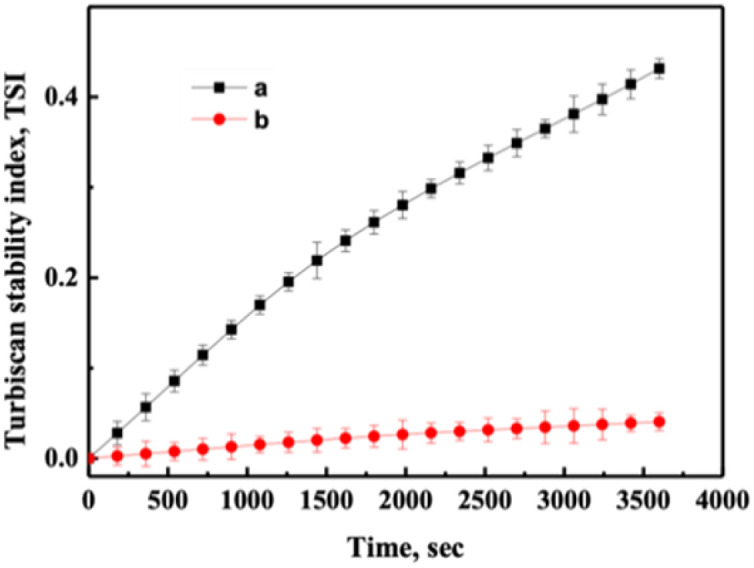
Effect of DESs on the stability of CNF. (a) CNF and (b) 1-3-1-2 h.

Fig. 7 and S5† show TSI diagram of CNF samples, from which it could be found that the TSI value of samples not pretreated by DES was the largest at the same time. Comparing Fig. 7a with 7b, it could be found that the TSI value of the samples obtained was smaller with the prolongation of DES pretreatment time, which means the samples were more stable. The samples remained stable after one week (Fig. S6†).

**Fig. 7:**
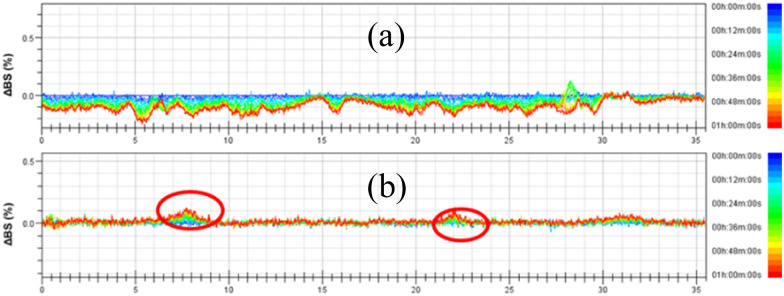
TSI as a function of time for samples of CNF. (a) CNF and (b) 1-3-1-2 h.

Fig. 8 and S7† show thermogravimetric (TG) curves of CNF–zeolite composite films and zeolite powder, respectively. According to the calculation in Fig. S7,† the maximum adsorption capacity of zeolite powder for thiol was 33.3 mg g^−1^. As shown in Fig. 8, with the increase in the CNF content, the adsorption capacity of thiol for CNF–zeolite composite films shows a downward trend. When the amount of CNF was 20 wt%, the adsorption capacity of the membrane for mercaptan was 22 mg g^−1^, and when the amount of CNF reaches 50 wt%, the adsorption capacity of the membrane for mercaptan decreases to 9.2 mg g^−1^.

**Fig. 8:**
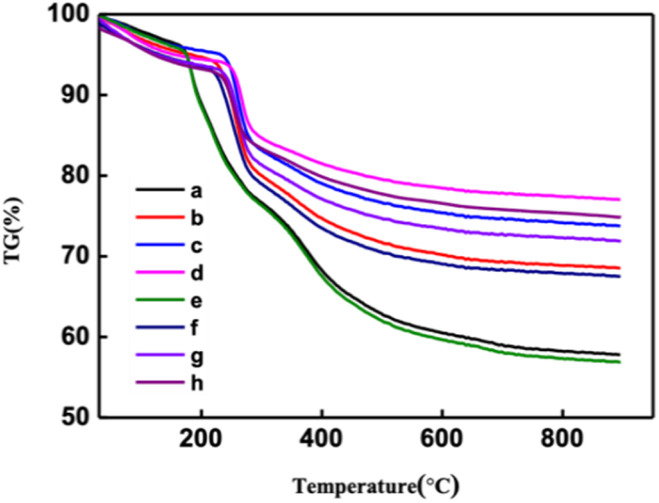
Uptake of thiols with CNF–zeolite composite films: (a–d) as received. 1–1, 1–2, 1–3 and 1–4, respectively. (e–h) after exposure to ethanethiol for 1 h. 1–1, 1–2, 1–3 and 1–4, respectively.

Fig. 9 shows examples of the resulting free-standing and flexible CNF-zeolite films after vacuum drying in an oven at a temperature of 35 °C and a pressure of −0.6 bars for 24 h. As shown in the TGA curves in Fig. 8, some water (less than 4 wt%) remains in the CNF–zeolite films prepared with ZSM-5 after drying. The SEM micrograph of CNF–ZSM-5 (Fig. 9b) shows that the ZSM-5 zeolite particles are homogeneously distributed in the CNF network.

**Fig. 9:**
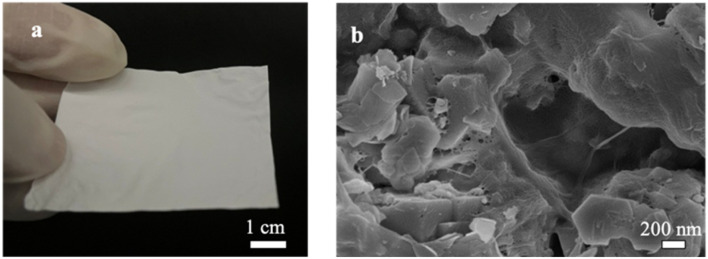
Free-standing CNF–zeolite (1–4) films: (a) photograph of CNF–zeolite free standing films. (b) SEM micrograph of CNF–ZSM-5 top surface zeolite particles entrapped and connected by the cellulose network.

Fig. 10 shows the tensile strength of the composite CNF–ZSM-5 films. The strength and flexibility are primarily determined by the CNF network and the CNF content must be sufficiently high to enable the nanosized fibrils to form a percolating network.^31^ The tensile strength of the composite CNF–ZSM-5 films reaches values of 4.6 MPa and higher when the CNF content is above 20 wt%. Hence, the mechanical measurements strongly suggest that it is the CNF network that controls the mechanical properties and because CNF is such a strong material, it is also possible to produce strong films at zeolite contents of 80 w/w% and above.

**Fig. 10:**
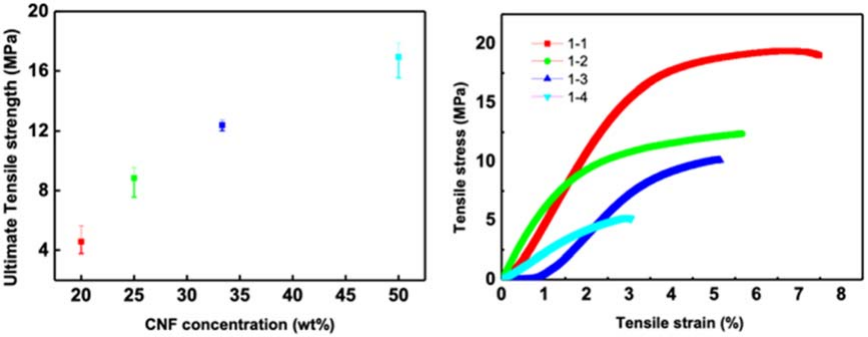
Mechanical properties of zeolite–CNF composite films.

In summary, we developed a novel method for directly sulfated cellulose nanofibers. Compared with other DESs used in sulfation modification, DESs used in this experiment could be prepared at lower temperature (60 °C), and the reaction temperature (100 °C) during modification was also lower. The results showed that the partial sulfonation of cellulose increased the fibrillation trend of fibers and made the length–diameter ratio of sulfated CNF fibers higher than that of non-sulfated fibers. Under the same mechanical treatment times, the energy consumption of fibers pretreated by DESs (1.56 × 10^7^ kJ kg^−1^) is reduced by 83.58% compared with the unpretreated pulp (9.50 × 10^7^ kJ kg^−1^). In addition, low-toxic chemicals that are easy to handle and environmentally friendly were used, and no external solvents were used. The recovery rate of DESs was 69–80%. This method is a potential way to obtain nanocellulose even on a large scale.

## Author contributions

Writing – original draft: Guangrui Ma, investigation: Guangrui Ma and Zhiguo Zhang, supervision: Ming He and Jiachuan Chen, and conceptualization and project administration: Guihua Yang and Ming He.

## Conflicts of interest

The authors declare no competing financial interest.

## Supplementary Material

NA-005-D2NA00769J-s001
