# Supplementary material for: Facile sulfation of cellulose via recyclable ternary deep eutectic solvents for low-cost cellulose nanofibril preparation
Source: Nanoscale Adv. 2022 Nov 21;5(2):356–60. doi: 10.1039/d2na00769j (PMC9846476; doi:10.1039/d2na00769j)
Supplement: NA-005-D2NA00769J-s001 [file NA-005-D2NA00769J-s001.pdf]

Electronic Supplementary Material (ESI) for Nanoscale Advances.
This journal is © The Royal Society of Chemistry 2022

Supplementary Information

Experimental Section

Materials.

Bleached hardwood kraft pulps (Eucalyptus, Brazil) were used as a starting material and were provided as dry sheets. sulfamic acid (95.0%) was obtained from Shanghai Macklin Biochemical Co., Ltd (China), Urea (≥ 99.0%) and Choline chloride were obtained from Sinopharm Chemical ReagentCo., Ltd (China), copper-ethylene diamine (CED)-solution from China National Pulp and Paper Research Institute (China). ZSM-5 powder (MFI type, cation: sodium, Si/Al: 76~80, particle size ~ 6.5 μm, Macklin Int. China). The low molecular weight thiols, Ethanethiol (T_b= 35 °C, Aladdin Int, China) was used as the target odor. $CaCl_2$ and polyethylene glycol (PEG, Aladdin Int, China, Mw=8000 g/mol) were used as-received. Deionized water (Conductivity=1.0 us/cm, 25 °C) was used in all experiments.

Fiber treatment with the DES.

The fiber treatment with the DES was performed in a similar manner described previously.[1,2] The components of DES were mixed together using amagnetic stirrer in an oil bath at 60 °C at a molar ratio (sulfamic acid: urea: Choline chloride) of 1:3:1. After obtaining a clear liquid (at around two hours), the 5.5%wt of cellulose was added (Mass ratio of cellulose to DES). The suspension was mixed with a magnetic stirrer, then, the temperature of the system was increased to 100 °C (heating speed at around 5 °C/min), and the reaction was allowed to proceed for desired time (30, 60, and 120 minutes). The reaction mixture was then removed from the oil bath and allowed to cool for 5 min. The reaction was finally terminated by the addition of excess of water, followed by filtration and washing with water until the filtrate was neutral. The washed fibers were collected and stored at 4 °C until further use. The mass yields of the samples were 80%–99%, and the yield losses were mainly attributed to sample handling (e.g., small amounts of the fibers remained on the filter paper).

Analysis of the fiber dimensions.

Two milligrams of the fibers (either original cellulose or DES treated fibers) were manually mixed with water to obtain a 0.004 wt% suspension. A 50 mL aliquot of the suspension was prepared and then analyzed using a Fiber image analyzer (Valmet Fiber Image Analyzer, Valmet FS5, Finland). The fiber width, length (Lc (w)) and curl were obtained as average values of over 5000 individual qualified fibers captured by the scanning camera in the instrument.

Elemental analysis of sulfated cellulose.

The samples were dried in an oven at 60 °C overnight. The nitrogen, carbon, hydrogen and sulfur and sulfur contents of the samples were analyzed using the elemental analyzer (Vario EL II, Germany), respectively. The degree of substitution (DS) was calculated using Eq. 1:[3]

$$DS = \frac{S \times 162.15}{3206 - (S \times 910)} \qquad (1)$$

where S is the sulfur content, 162.15 mmol/g is the molecular weight of the anhydroglucose unit, and 97.10 mmol/g is the molecular weight of the ammonium sulfate group.

Degree of polymerization.

The average degree of polymerization (DP) of original cellulose pulp, DES-treated cellulose, and nanofibrillated celluloses was evaluated from the limiting viscosity, measured in CED solution according to the ISO 5351 standard. The analysis was conducted in triplicates, and the results were averaged. Samples were freeze-dried prior to measurement. The limiting viscosity values were converted to DP values using the following Eq. (2):

$$DP = \left(\frac{1.65[\eta] - 116H}{C}\right)^{1.111} \quad (2)$$

Where [η] is the limiting viscosity, C is the mass fraction of cellulose, and H is the mass fraction of hemicellulose.

This calculation corrects the contribution of hemicellulose to the limiting viscosity value and DP of cellulose, assuming that the average DP of hemicellulose is 140.[2]

Diffuse reflectance infrared Fourier transform (FT-IR).

The original and DES-treated pulp was measured after drying at 60 °C. The spectra were recorded using Fourier Transform Infrared Spectrometer (Bruckvertex 70, Germany). The number of scans was 32 and the resolution was 4 cm^{-1}.

Field emission scanning electron microscope (FE-SEM).

The DES-treated sample was weighed, to match 2.0 mg of the absolutely dry mass, into a decanting glass and then diluted with 200 ml of water. Then the sample was dried via vacuum filtering on a polyvinylidene fluoride membrane with a pore size of 0.45 μm, which was then dipped in liquid nitrogen and dried with a freeze-dryer. When the samples were dry, a double-sided sticker was glued on the bottom of the weight, and a small piece of the film sample was glued on the sticker. Prior to the measurement, the samples were coated with gold. Field-emission scanning electron microscopy (FE-SEM) was conducted with a SEM (Hitachi Regulus® Regulus 8220, Japan) working at a low acceleration voltage (5 kV) and short working distance (8.0 mm).[3]

Nanofibrillation of cellulose.

Pulp samples were disintegrated with a laboratory disintegrator at 1% consistency for 5 min before fibrillation. The DES-treated pulp was fibrillated once by a grinder (Supermasscolloider MKCA6-5J, Masuko Sangyo, Japan). The grinder is equipped with a power meter to record electrical energy input. Pulp feeding was achieved by gravity. The rotational speed was set to 1800 rpm, and the gap of the two disks was adjusted to −150 μm from motion zero position after pulp was loaded. The fibrillated pulp suspension was discharged by centrifugal force.[5] Each sample passed the system 1 pass. Then nanofibrillated with Microfluidics' M-110EH-30-microfluidizer. Each sample passed the system 5 passes through 200 μm chambers at 200 bar.

Atomic Force Microscopy (AFM).

AFM measurements were carried out using a Dimension Icon (Bruker, Multimode8, Germany). Substrates were prepared by casting polyoxyethylene (PEO) solution (20% by mass) onto a freshly cleaved mica substrate. After 10 min, the substrate was blown dry. Diluted aqueous nanocellulose suspensions (ca. 0.01% by mass) were deposited onto the PEO-coated mica disks. The sample was air dried naturally. Topographical imaging was performed in Scanasyst tapping mode using a Scanasyst-air tip.

Determination of surface charge density.

The surface charge density of SCNFs was determined by polyelectrolyte titration with particle charge detector (BTG Mu¨tek PCD-03, Germany).[2]

Energy Consumption.

The energy consumption in processing the pulp dispersion in the microfluidization process was estimated by Eq. (4):

$$E_m = \varepsilon \frac{V \times \rho}{1000} \left(\frac{V}{V_{micro}} \times t_{res} \times N \right) \qquad (4)$$

where $\varepsilon = 1.0 \times 10^7$ W/kg is the turbulent energy dissipation rate attainable in the micro-mixing chamber of M-110EH-30, which has a volume of $V_{micro} = 1.0 \times 10^{-6}$ ml. $t_{res} = 1.0 \times 10^{-6}$ s and N are respectively, the residence time of the dispersion in the micro-mixing chamber and the number of circulation passes being applied. The numerical values of ε, V_{micro}, and t_{res} were taken as disclosed by the Microfluidics Corp[4,5].

Recycling the DES.

Water was removed from the DES-water solution with EYELA rotavapor using approximately vacuum of −1.0 mbar and water bath at 60 °C. Evaporation was conducted until no water was observed to drop into collection flack. After evaporation, the mass of the DES was measured. After calculating the yield, DES was reused after supplementing the consumed sulfamic acid.

Thermogravimetric (TG) analysis.

The freeze-dried cellulose nanofibrils were ground into powder, and the samples were measured by thermogravimetric analyzer (TGA Q50, TA, USA) at a temperature rising rate of 10 °C min^{-1} under nitrogen protection, and the temperature range was 30~900 °C.[6]

Stability analysis.

The stability was studied using an optical analyzer (Turbisoft LAB Ageing Station, Formulaction Inc). In this experiment, CNF samples were placed in a cylindrical glass cell followed by the Turbiscan at 25 °C for 1 h. The stability of each sample was assessed based on the variation of transmission (ΔBS). A Turbiscan Stability Index (TSI) was calculated according to Eq. (1):

$$\text{Turbiscan Stability Index (TSI)} = \sqrt{\frac{\sum_{i=1}^{n} (x_i - x_{BS})^2}{n-1}} \qquad (1)$$

Where x_i=Average backscattering for three minutes measurement; x_{BS}=Average value of x_i; n =the number of scans[7].

Preparation of CNF-Zeolite Composite films.

SCNF (1-3-1-2h) dispersions with a concentration of 0.5 wt% were prepared by dispersing 1 wt% sulfated cellulose nanofibrils (CNF) in millipore water by stirring (IKA T25, IKA-Werke GmbH & Co.KG) for 15 min and sonication in an ultrasonic bath for 30 min. Dilute aqueous dispersions of the de-agglomerated zeolite particles with a concentration of 0.5 wt% was prepared and mixed with 0.01 wt% polyethylene glycol and 13 mmol/L of $CaCl_2$. Homogeneous composite dispersions of zeolite and CNF were obtained by mixing the CNF dispersion (0.5 wt%) with a zeolite dispersion (0.5 wt%) that were sonicated and de-aired. Free standing films were formed by vacuum filtration of the Ca-ion crosslinked zeolite-CNF dispersion using filter papers with pore diameter of 2 μm. Most of the water was removed by treating the films in a vacuum drying oven (Blue pard, Shanghai Yiheng Scientific Instrument Co., Ltd., China) under a temperature of 35 °C at a pressure of -0.6 bars for 24 h. Zeolite

contents of zeolite-CNF free standing films are reported based on weight percentage of dry constituents with respect to the total initial dry solid weights excluding $CaCl_2$. For comparison, free standing ZSM-5-CNF films with CNF to ZSM-5 mass ratios were prepared by 1:1, 1:2, 1:3 and 1:4, respectively.

Characterization of CNF-Zeolite Composite films.

Thermogravimetric analysis (TGA Q50，TA, USA) was used to measure the amount of water and thiols adsorbed on the zeolite powders. The temperature was increased at a rate of 20 °C/min from room temperature to 900 °C under a flow of dry air. The maximum thiol uptake was determined by exposing zeolite powders (20 mg) in a sealed polyethylene tube containing 100 µl of liquid thiols at the boiling temperature of the thiols for 3 days. The zeolite powders were removed from the tube and exposed to air for 3 days to remove loosely attached thiols prior to TGA measurements. The difference between the total weight loss of zeolites at 900 °C after and before exposure to thiols is reported as the amount of thiols adsorbed on each respective zeolite powder after subtracting the weight loss due to water evaporation. The mechanical strength of the zeolite-CNF films were measured by uniaxial tensile tests on an Instron 5944 with a load cell of 50 N. The tensile tests were performed on rectangular films (35 mm by 10 mm) at 10% strain/min.

Table S1 physical property of fibers

	Fiber length/mm	Fiber width/µm	Curl/%	DP
1-3-1-0.5h	0.63	13.82	16.59	1071
1-3-1-1h	0.58	13.92	12.82	782

Table S2 Elemental analysis and determination of surface charge density of samples

	N	C	H	S	DS	Charge density$_{CNF}$
Original pulp	0	41.95	6.74	0	0	-
1-3-1-0.5h	1.01	39.9	6.02	0.97	0.05	0.25
1-3-1-1h	5.6	35.71	6.05	2.21	0.12	0.65
1-3-1-2h	7.21	34.45	6.43	2.62	0.144	0.80

Table S3 Energy Consumption of Samples

	Original pulp	1-3-1-0.5h	1-3-1-1h	1-3-1-2h
Energy consumption $\times 10^7$(KJ/Kg)	9.50	1.91	1.63	1.56

Table S4 The recovery rate of DES

DES	1-3-1-0.5h	1-3-1-1h	1-3-1-2h
Recovery rate/%	80.24	73.89	69.21

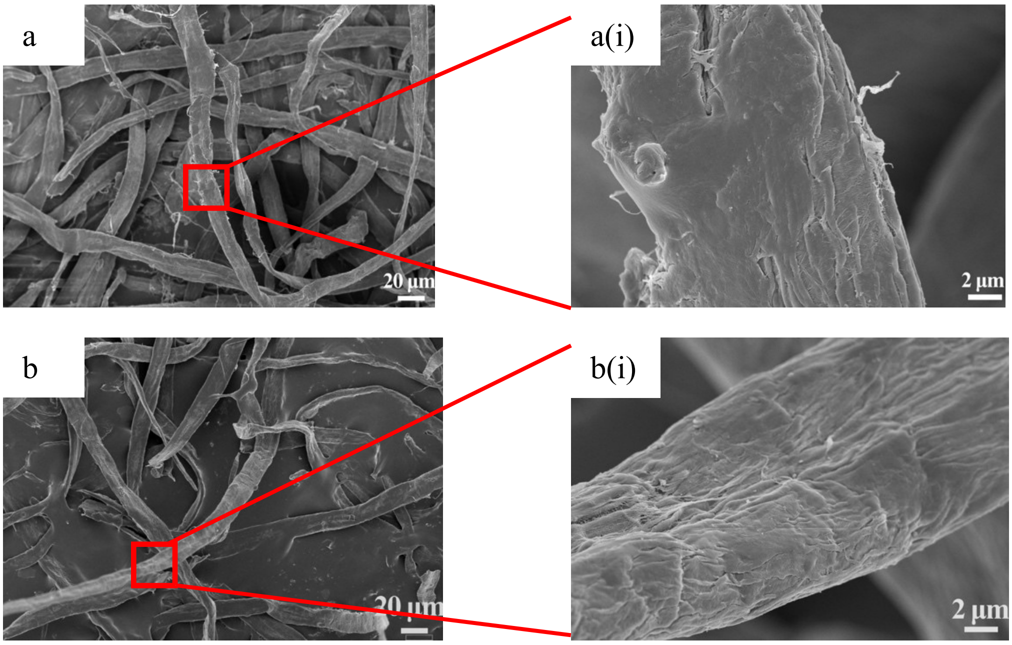

Figure S1: Scanning electron micrograph of fiber. a. 1-3-1-0.5h, b 1-3-1-1h

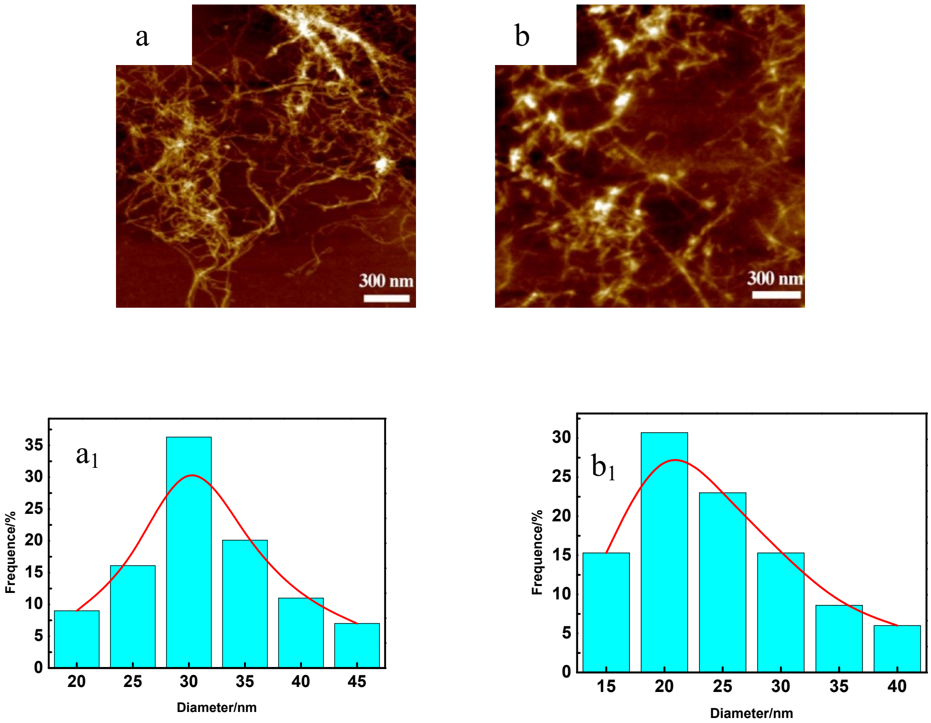

Figure S2: Atomic force microscopy of CNF samples. a. 1-3-1-0.5h, b.1-3-1-1h.

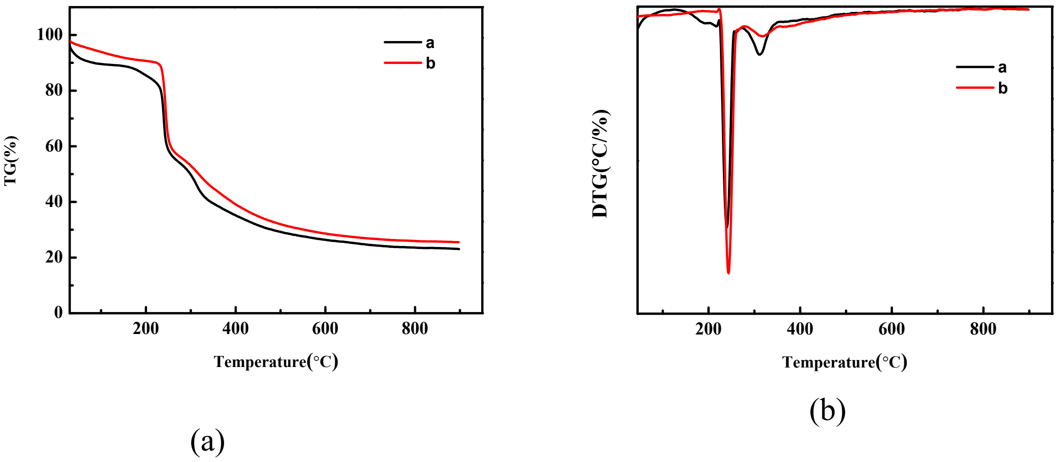

Figure S3: A Thermal stability spectrogram of CNF samples. a. 1-3-1-0.5h, b.1-3-1-1h.

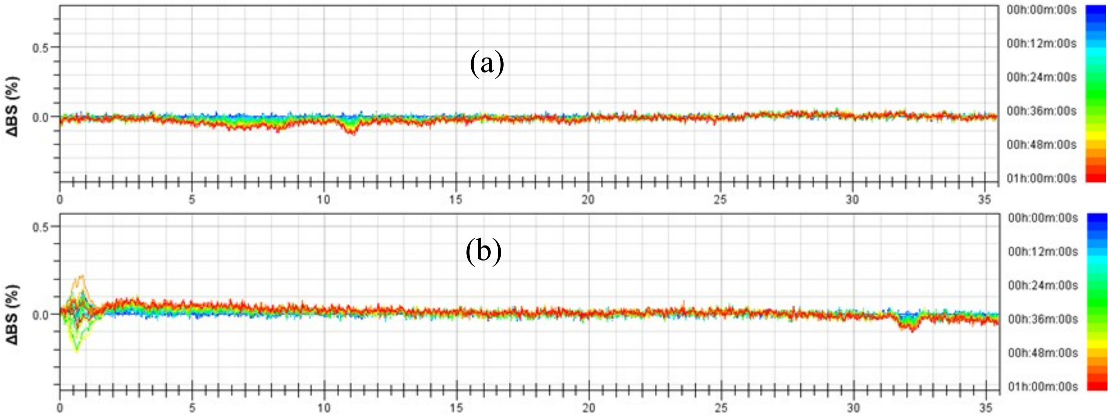

Figure S4: Effect of DES on the stability of CNF (measured by Turbiscan LAB Stability Analysis Tester within 1 h, the lines of different colors represent the changes in the light transmittance of the CNF suspension at different times) a. 1-3-1-0.5h, b.1-3-1-1h.

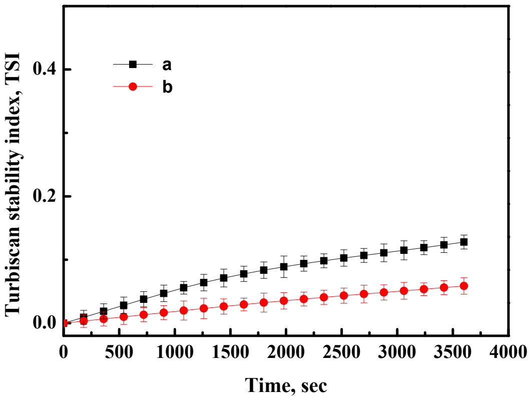

Figure S5: TSI as a function of time for samples of CNF. a. 1-3-1-0.5h，b. 1-3-1-1h.

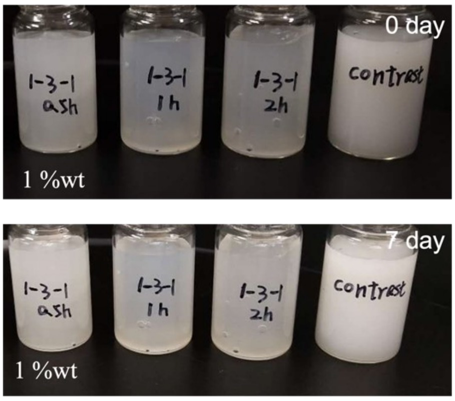

Figure S6: Sample sedimentation experiment

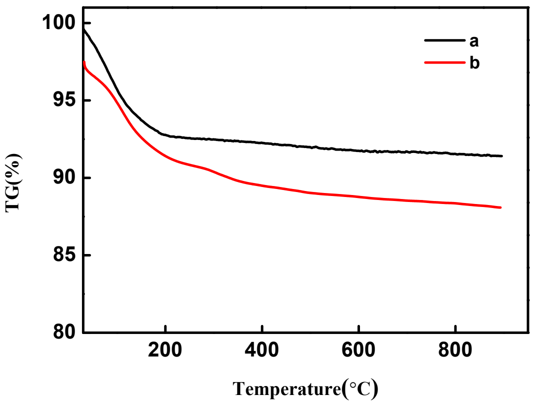

Figure S7: Uptake thiols with ZSM-5: Thermogravimetric (TG) curves of ZSM-5 powders; (a) as received; (b) after exposure to ethanethiol for 1h. The powders were not degassed before exposure to the thiols.

References:

1 J. A. Sirviö, M. Visanko and H. Liimatainen, *Green Chem.*, 2015, **17**, 3401–3406.

2 J. A. Sirviö, J. Ukkola and H. Liimatainen, *Cellulose*, 2019, **26**, 2303–2316.

3 K. Missoum, J. Bras and M. N. Belgacem, *Biomacromolecules*, 2012, **13**, 4118–4125.

4 B. R. Pearson, P. Å. Krogstad and W. Van De Water, *Phys. Fluids*, 2002, **14**, 1288–1290.

5 S. Luo, T. Liu and B. Wang, *Carbon N. Y.*, 2010, **48**, 2992–2994.

6 N. Keshavarzi, F. Mashayekhy Rad, A. Mace, F. Ansari, F. Akhtar, U. Nilsson, L. Berglund and L. Bergström, *ACS Appl. Mater. Interfaces*, 2015, **7**, 14254–14262.

7 W. Im, S. Lee, A. Rajabi Abhari, H. J. Youn and H. L. Lee, *Cellulose*, 2018, **25**, 3873–3883.
